# A qualitative exploration of the barriers and facilitators to self‐managing multiple long‐term conditions amongst people experiencing socioeconomic deprivation

**DOI:** 10.1111/hex.14046

**Published:** 2024-04-16

**Authors:** Abi Woodward, Danielle Nimmons, Nathan Davies, Kate Walters, Fiona A. Stevenson, Joanne Protheroe, Carolyn A. Chew‐Graham, Megan Armstrong

**Affiliations:** ^1^ Research Department of Primary Care and Population Health University College London London UK; ^2^ School of Medicine Keele University Keele UK; ^3^ Wolfson Institute of Population Health Queen Mary University of London London UK

**Keywords:** accessing healthcare, multiple long‐term conditions, qualitative, self‐management, socioeconomic deprivation

## Abstract

**Background:**

Globally, it is estimated that one in three adults live with two or more long‐term conditions (multiple long‐term conditions, MLTCs), that require self‐management. People who experience socioeconomic deprivation face significant health inequalities due to a range of interrelated characteristics that lead to a lack of resources and opportunities. Previous research with underserved populations indicate low levels of trust towards primary care providers and potential barriers for developing patient‐healthcare professional relationships. The purpose of this paper is to explore the barriers and facilitators to self‐managing MLTCs, amongst people who experience socioeconomic deprivation.

**Methods:**

Semistructured one‐to‐one interviews with adults (*n* = 28) living in London and Sheffield, United Kingdom with MLTCs who are experiencing socioeconomic deprivation. Participants were recruited through general practices, community channels and social media. Data were analysed in NVivo using reflexive thematic analysis methods.

**Findings:**

Four analytical themes were developed: (1) challenges in accessing healthcare services, financial assistance, and cultural awareness; (2) empowerment and disempowerment through technology, including digital exclusion, and use of technology; (3) impact and causes of exclusion on self‐management, including social isolation, area‐based and economic exclusion, and health‐related stigma and (4) adapting self‐management strategies, including cost‐effective, and culturally/lifestyle appropriate strategies.

**Conclusions:**

Future health interventions and services need to be developed with consideration of the combined complexities of managing MLTCs while experiencing socioeconomic deprivation. Increased awareness in practitioners and commissioners of the complexities surrounding the lives of people experiencing socioeconomic deprivation, and the need for targeted strategies to promote self‐management of MLTCs are of great importa.

**Patient or Public Contribution:**

A patient advisory group contributed to all stages of the study, including providing important feedback on study documents (topic guides and recruitment materials), as well as providing critical insights surrounding the interpretation of interview data.

## 1 INTRODUCTION

It is estimated that one in three adults worldwide live with two or more long‐term conditions (multiple long‐term conditions, MLTCs), that require ongoing management, including self‐management.^1^ Populations living in more deprived areas develop MLTCs earlier compared to those living in more affluent areas,^2^ and across England, people living in deprived areas die earlier than those in more affluent areas.^3^ The burden of MLTCs in England is increasing, and persistent inequalities due to MLTCs are found to be worse in working‐age adults and for those with complex MLTCs (e.g., four or more long‐term conditions).^4^ People who experience socioeconomic deprivation face inequalities in health due to a range of interrelated characteristics that lead to a lack of resources and opportunities,^5^, ^6^ including low or limited social capital. The concept of ‘social capital’ broadly refers to the resources and benefits that individuals and groups gain from their social networks, relationships and interactions.^7^ As such, this study draws upon the principles underpinning the Burden of Treatment Theory to highlight that the capacity to manage conditions is not simply due to people's own ability but also depends on their social skills and social capital.^8^

Globally, health inequalities have been increasing in many countries, and it is suggested that ‘wide‐ranging, long‐term policy programmes that simultaneously target multiple social determinants of health’ are needed to reduce health inequalities.^9^ The long‐term nature of many health conditions results in individuals being responsible for managing their own health (i.e., self‐management).^10^ According to Corbin and Strauss's framework, self‐management involves (1) medical management; (2) changing unhealthy or maintaining healthy behaviours and (3) emotional management.^11^ Self‐management strategies include the person managing symptoms and treatment, the psychological impacts and lifestyle changes required for their conditions.^12^ Health literacy, culture, economic status and other contextual factors all influence individual self‐management behaviours.^13^ Effective self‐management strategies or interventions are associated with improved health outcomes, yet self‐management interventions are shown to be less effective in people experiencing socioeconomic deprivation.^10^

Previous research with underserved groups, including those experiencing different forms of deprivation, and people from minority ethnic backgrounds, indicate low levels of trust towards primary care providers^14^ and systemic racism.^15^ People experiencing socioeconomic deprivation have reported feelings of not being heard and believing clinicians do not understand the context of their lives,^14^ which may lead to barriers for developing patient‐healthcare professional (HCP) relationships.^16^

A systematic review conducted by the authors found that aspects in people's lives that can facilitate self‐management include; opportunities to maintain independence, being in paid employment, receiving informal support from family and peer networks, engaging in physical activity and eating a healthy diet.^6^ In a further systematic review focused on self‐management interventions tailored for people experiencing socioeconomic deprivation, the trials on MLTCs showed promise in improving outcomes but have not been adopted into practice, suggesting implementation barriers.^17^ The most effective tailoring of self‐management interventions included adaptions for low literacy, the involvement of community health workers or peer leaders, providing helpful materials if needed and financial incentives.^17^ There is however limited evidence from UK‐based studies on the impact of socioeconomic deprivation on self‐management.^6^ More research is needed to better understand individual deprivation, including the way in which individuals manage the intersecting components surrounding their MLTCs, self‐management and challenges associated with socioeconomic deprivation.^6^, ^17^, ^18^

The aim of this paper is to explore the barriers and facilitators to self‐managing MLTCs, among people who experience socioeconomic deprivation.

## 2 METHODS

### 2.1 Design

Qualitative study using semistructured interviews.

### 2.2 Patient and public involvement and engagement (PPIE)

A patient advisory group (PAG) contributed to all stages of the study, including providing important feedback on study documents (topic guides and recruitment materials), as well as providing critical insights surrounding the interpretation of interview data.

### 2.3 Study population and setting

The setting for the research was London and Sheffield, England. Eligible participants were adults (18 years and over), diagnosed with two or more long‐term conditions (MLTCs) who lived in an area of deprivation (categorised as the bottom 20% by the Index of Multiple Deprivation [IMD]). Due to the area of deprivation being insufficient alone to determine individual characteristics of socioeconomic deprivation, potential participants were also asked to ‘self‐identify’ with one of the following statements:

1. I have had no further education or training beyond the age of 16.
2. I feel like I am just about getting by or have difficulties affording the necessities I need in daily life such as housing, heating, food, clothing or access to the internet.
3. I am currently unemployed, looking for work or earning lower wages than I need to get by.

The statements were formulated in consultation with the PAG and support the exploration of data in an intersectional way to see how different socioeconomic factors interact with each other. Interviews were conducted according to participants' preference, for example, face‐to‐face in participants own home or remotely via Zoom/Microsoft Teams/telephone. The study excluded people who:

1. Were currently hospital inpatients.
2. Did not have the capacity to consent.
3. Had less than 6 months to live (due to the complexity of their needs).
4. Could not speak English sufficiently to participate in an interview in English.

### 2.4 Sampling and recruitment

Purposive sampling supplemented with snowballing techniques^19^ was used. Participants were recruited through general practitioner (GP) surgeries located in areas of deprivation (IMD > 20%). Participating practices conducted electronic database searches of patient medical records and sent out postal invitations to potential participants. Other means of recruitment used community channels such as voluntary/community sector organisations/groups and social media. Recruitment flyers were used to assist with participant identification. The study team was supported by Noclor, a Clinical Research Network support service with good links to practices with patients experiencing socioeconomic deprivation. All potential participants were screened over the phone/email by the lead author (A. W.) for eligibility using the criteria set out above. Participant characteristics are detailed in Table 1.

**Table 1:** Participant characteristics.

Category	Number of participants
Recruitment location	Greater London, *n* = 21 Sheffield, *n* = 7
Recruitment route	GP practice, *n* = 10 Other (e.g., voluntary/community organisations and groups, social media), *n* = 18
Gender	Male, *n* = 12 Female, *n* = 16
Age range	20–90 years (average age 55)
Ethnicity (as identified by individual participants)	White British, *n* = 10 White Irish, *n* = 3 Italian Muslim (other White), *n* = 1 White American, *n* = 1 European‐North African, *n* = 1 South Asian, *n* = 8 (variances included: Bangladeshi, British South Asian, British Asian, South Asian, Indian, Pakistani Muslim, British Pakistani) Black African, *n* = 3 Afro‐Caribbean, *n* = 1
Long‐term conditions reported	Physical only, *n* = 11 Mental only, *n* = 1 Combination of physical and mental, *n* = 16 Average number of conditions per participant, *n* = 5 (range 2–12) Participants with complex MLTCs, (e.g., 4+) *n* = 18
Further education beyond the age of 16 years	Yes, *n* = 20 No, *n* = 8
Employment status	Employed part‐time, *n* = 6 Employed full‐time, *n* = 2 Unemployed, *n* = 2 Unable to work—long‐term sick/disabled, *n* = 10 Looking after family/home, *n* = 1 Retired, *n* = 7 (includes retired on grounds of ill health, *n* = 1)
Housing status	Rented (private), *n* = 4 Rented (social housing), *n* = 14 Owner occupier, *n* = 5 (includes shared ownership, *n* = 1) Living at home with parents, *n* = 4 Temporary accommodation, *n* = 1
Estimated household income	<£15,000, *n* = 14 £15,000–19,999, *n* = 7 £20,000–29,999, *n* = 3 £30,000–39,999, *n*= 1 £40,000–49,000, *n* = 2 (both based on parents' income) £50,000–59,000, *n* = 1

Abbreviations: GP, general practitioner; MLTC, multiple long‐term condition.

### 2.5 Data collection

Semistructured interviews were conducted by the lead author between March 2022 and January 2023 which allowed for flexible and open‐ended questions.^20^ Participants provided written or verbal (audio‐recorded) informed consent and were offered a £50 voucher for participation as recommended by the PAG. A topic guide (see Supporting Information S1: Appendix 1) was developed alongside the study's PAG and research advisory group. The topic guide was based on the self‐management taxonomy^21^ and the principles of self‐management as outlined by Corbin and Strauss.^11^ All participants were provided with a Participant Information Sheet and offered the chance to ask questions. All interviews were audio‐recorded using a password protected digital recorder or via a virtual meeting platform. Each participant completed a short socioeconomic demographic form with the researcher before the interview.

### 2.6 Data management and analysis

Audio recordings were transcribed verbatim and checked for accuracy by the lead author. Interview transcripts were anonymised, imported into NVivo and analysed using an inductive reflexive thematic analysis (TA) approach. In reflexive TA, themes are understood as actively created by the researcher at the intersection of the data and the researcher's interpretive framework, acknowledging researchers' assumptions.^22^ Reflexivity is therefore essential to the successful implementation of TA and includes reflecting on the assumptions of the researcher(s). This study takes an *experiential* approach to qualitative research which refers to the emphasis on understanding phenomena through individuals' experiences within their social and cultural contexts.^23^ Initial coding was created by A. W. and themes developed in consultation with other authors (M. A., D. N., N. D.), then reviewed and refined by the wider team (K. W., F. S., A.‐C. C. G., J. P.). A. W. is an experienced qualitative researcher, M. A. was a child carer for someone with MLTCs experiencing deprivation. D. N. provided insights from her own ethnic background which was important given the diversity of the research sample. The multidisciplinary expertise of the research team provided perspectives from researchers with backgrounds in psychology and sociology and practicing GPs with expertise in self‐management and health inequalities.

## 3 FINDINGS

Analytical themes and subthemes (see Table 2) were developed in line with reflexive TA (see Section 2). These form the basis for the presentation of data which takes an intersectional approach to describing the multiple dimensions of socioeconomic deprivation and the impact on self‐management of MLTCs.

**Table 2:** Analytical themes.

Analytical themes	Subthemes
Challenges in accessing services	▪ Healthcare services ▪ Navigating financial assistance ▪ Lack of cultural awareness in health services
Empowerment and disempowerment through technology	▪ Digital exclusion ▪ Technology to self‐manage and self‐educate
Impact and causes of exclusion to self‐management	▪ Social isolation and low social capital ▪ Area‐based exclusion ▪ Economic exclusion ▪ Health‐related stigma
Adapting self‐management strategies	▪ Cost‐free and low‐cost activities ▪ Culturally and lifestyle appropriate strategies

The challenges that participants with MLTCs faced, alongside experiences of socioeconomic deprivation, impacted their ability to self‐manage. Due to the in‐depth nature of the interviews, participants naturally highlighted some challenges that are well documented in literature on MLTCs.^6^, ^24^, ^25^ It is contextually important, however, to briefly outline these known challenges since they were shown to be prevalent amongst socioeconomically deprived groups and were barriers to their self‐management. For instance, having the time to discuss specific conditions with a GP was important yet many participants felt the time allocated during their appointment was adequate only for discussing single conditions rather than MLTCs. Challenges were reported around management of certain conditions being prioritised during appointments, leading to a lack of patient autonomy. The interlinking nature of MLTCs meant that physical conditions, for example, chronic pain, could exacerbate mental health conditions such as anxiety and depression, as well as leading to an overlap in symptoms. Illustrative data extracts are given below with interview identifiers.

### 3.1 Challenges in accessing services

#### 3.1.1 Healthcare services

Challenges surrounding access to health services were reported by participants. For some, the area they lived in and where their general practice was located was problematic. Several participants spoke about the negative consequences of relocating due to a change in circumstances, as explained below:

When I lived at [more affluent area], I was with the [GP surgery], now that's been consistently rated as the top GP surgery in [City] for four years running … I moved here [to area of deprivation], I'm on a different surgery, and it's like, I mean a bit of a backwater. It's very difficult to get hold of anybody. (P16_male_White British_57 years)

Participants highlighted their awareness of health services that may benefit them, either due to their own research or word‐of‐mouth. Many were not, however, able to use the information acquired about their conditions or additional services to make decisions about their own health, and relied on their GP to relay important information:

I've heard that there's something called a social prescriber, because at the Community Centre there was two there … And I'm thinking, ‘What do they do? How can they help me?’ … But I don't know if my GP does that. Because my GP is not going to spend time talking to me, like you know, about things that I need to find more information on. (P14_female_British Asian_57 years)

In contrast, some participants reported high levels of self‐reliance in their approach to managing their MLTCs, which was typically due to a response to challenges experienced previously within the heath system:

I asked to see the neuropsychiatrist, I asked for referrals to manage medication various other things even on a social thing it just feels like there needs to be more of a connection between the health service and other public services and even charities. I feel like a lot of the stuff I've discovered or managed to gain hold of is all through personal research rather than being guided by the medical service… (P07_female_Indian_49 years)

#### 3.1.2 Navigating financial assistance

Participants reported a variety of challenges relating to the practicalities involved in navigating financial assistance. The UK cost‐of‐living crisis, which refers to people experiencing rising inflation while household incomes fall, was mentioned frequently during the interviews in relation to financial precarity. Several participants who, due to having MLTCs, were either in receipt of state benefits or had tried to access them, said that lack of money contributed to anxiety and worries:

I do get scared, very scared, because money is limited for me … but it's going to be always. I don't have no savings, nothing. And I do worry about my benefits because I sometimes think that they might stop my benefits… (P20_female_Pakistani Muslim_58 years)

The application process for the UK benefit, Personal Independence Payment (PIP) was said to be a barrier to accessing help and instead of facilitating self‐management, failed attempts could worsen mental health:

…people keep telling me I'm eligible for PIP … but for me it's really traumatic filling in the forms … they're a bit ambiguous and confusing, especially with people with hidden disabilities… (P07_female_Indian_49 years)

As such, PIP is designed to ease the financial strain of everyday life for those with a long‐term physical or mental health condition or disability, yet some participants who were in receipt of PIP, still experienced a financial strain due to the cost‐of‐living which negatively impacted mental health:

…it was through the … PIP Payment and I managed to kind of put a little bit away because I was basically just kind of prepping for the extra payment in bills… So now it's back to kind of squirreling and saving … when you're in a condition of depression, that's really difficult. You know, you can't afford to get stressed out totally because you could end up becoming unwell again. And I suppose I don't really want to reach the point of breakdown again… (P05_male_South Asian_37 years)

Many participants who were not eligible for free NHS prescriptions described being on low incomes and relying on medication to manage their conditions, which was costly:

I have to take a lot of medication, so whenever I buy the medication, it's cost me a lot… So that's affect me because when your income is low, every time GP changing your medication on something, add up on you … it's cost a lot, and medication is costly. (P03_female_Bangladeshi_38 years)

#### 3.1.3 Lack of cultural awareness in health services

Socioeconomic challenges were exacerbated amongst participants from minority ethnic groups. Some participants spoke about cultural or language barriers when visiting a GP, which could impact on their ability to seek information to facilitate self‐management:

Some people can speak English as a mother tongue, they can read and write … And a lot of people don't know, so when you give them English version, like … I'm from Bangladesh … Bangladeshi people, they don't know how to read English, but they can speak, [doctor] just give them the English leaflet. How they're going to do it? Do we think about that? No. (P03_female_Bangladeshi_38 years)

Interview participants described instances where miscommunications between themselves and HCPs arose, including assumptions that had been made about people's ethnicity, which impacted negatively on the patient–HCP relationship:

Maybe in the report he write something [like], ‘This Bangladeshi woman that grow up in Italy’, I never said that I'm Bangladeshi. Just because I have a hijab doesn't mean that I'm Bangladeshi. Just because I live in East London, I'm not Bangladeshi … I was so upset after I read this report, because this information in any case was not relevant for the report. (P10_female_Italian Muslim_37 years)

However, some participants did speak about positive experiences with HCPs and positive patient‐HCP relationships, suggesting ethnic background was not always a barrier to good, quality care:

…actually—a GP who was from—like English, national born and everything, and … the great thing about her she never really showed that, because you're like this, from this background, there's nothing else for you. You know what, let's try and see how everything goes. If you're not happy with it there might be other solutions. (P02_female_Black African_36 years)

### 3.2 Empowerment and disempowerment through technology

#### 3.2.1 Digital exclusion

Several participants reported having limited or no digital skills, making access to online and/or electronic health services challenging:

I've got so many texts [from NHS] … and I haven't got a clue… (P27_male_Afro Caribbean_66 years)

For others, technological challenges were combined with financial barriers:

…I do [have problems], but then I can't afford to get the internet and plus I don't know how to do it. (P20_female_Pakistani Muslim_58 years)

Participants felt having more digital skills could lead to greater opportunities for accessing health services, as well as offering more flexible employment options to work around MLTCs:

…it would be nice … to have a computer or something and be able to learn that or improve those [IT] skills … I think I'd be able to earn even a bit more money like, I don't know, working from home… (P14_female_British Asian_57 years)

Limited digital skills or a lack of digital access could result in exclusion from certain activities or services which impacted self‐management capabilities. While health apps could facilitate self‐management, some participants experienced usability issues, indicating that assistance with app functionality would be beneficial:

So I have been suggested this app [for diabetes]. I have downloaded it but I struggle with using the app, like to put in the calculation … it's quite challenging, it's quite difficult. It's not simple to use … so I haven't bothered to use the app. (P04_male_British South Asian_26 years)

#### 3.2.2 Technology to self‐manage and self‐educate

Many participants self‐educated themselves about their conditions and potential help, often through online resources. Those who did self‐educate, tended to display more proactive self‐management behaviours:

The internet. I can't think I've ever needed to go anywhere else. I did once start reading a book by said specialist about coeliac disease but it was so dry I lost interest [laughs]. I'm sure there was more information in there, but … I gave that up. (P13_male_White British_45 years)

Some participants were able to use apps to help monitor and/or keep track of their symptoms, or virtual assistant technology to set medication reminders:

I make sure Alexa has got my alarm sorted so that I take the drugs at the right time. And I found modern technology really great because Alexa will tell me it's time to take your meds… (P16_male_White British_57 years)

### 3.3 Impact and causes of exclusion to self‐management

#### 3.3.1 Social isolation and low social capital

Several participants highlighted that having MLTCs impacted negatively on their relationships with others:

I actually have no real life, like physical friends … 15 years ago when I was 20, I essentially lost all my real‐life friends … people just drop off as life moves on and you're stuck at home, so all my friends that I currently have are people I've met online… (P22_male_White British_35 years)

Another participant described perceiving their friends as having ‘compassion fatigue’:

… for a long time I hadn't really been very social … when they're [friends] asking—you're telling them what's happened but it feels like … a bit emotionally draining for them to have to listen to it all the time and you get the feeling that they're slowing drifting further away… (P07_female_Indian_49 years)

Some participants were identified as being at the extreme end of social isolation due to an absence of informal support or any positive relationships in their lives to help them manage:

…my family, my mum and dad aren't here. My brothers and sisters … they're not good, they're ignorant … they haven't helped me. Instead, they make me more upset, all of them … that makes me more, you know, worse, my health condition…. (P20_female_Pakistani Muslim_58 years)

Participants who had more opportunities to access informal support through family and/or friends and through more formal support structures, typically felt able to self‐manage their conditions better. Being housebound was extremely isolating, but some were fortunate to have family or friends in caregiving roles:

…managing my conditions is hard. I mean like I have my friends because the carers have stopped coming now, I couldn't afford them anymore basically, so family and friends do all the running around at the moment. Especially as my mobility scooter is not working … My friend or my brothers, whoever, they do the shopping and anything like that. Like I said I'm housebound… (P27_male_Afro Caribbean_66 years)

#### 3.3.2 Area‐based exclusion

Living in an area of deprivation could restrict access to support services or health provision that facilitated self‐management:

I used to go to the Parkinson's Trust [meetings] when I lived in [more affluent area] but since I moved here, I don't think they have a group … So, that was helpful back in the day, but I've been here four years and I haven't sought out any kind of support group. (P11_female_White American_67 years)

Levels of crime in residing areas could deter some participants from going out or accessing services:

I know that I feel quite lonely and I'd probably say quite isolated because I can't really do some of the things that a few of my friends can do …. I don't feel safe in the evenings, so I'm not able to kind of meet up with people. (P05_male_South Asian_37 years)

Access to provision that could facilitate or assist with self‐management could be impacted by mobility issues which were worsened by poor transport links. One participant, who was recommended to attend daily sessions at an NHS mental health facility, said:

Because I live quite far from them, you know, they sometimes say to me that you should come every day … to do sessions. But I can't because my mobility and plus I can't go there all the time … That's the problem because I don't live near, I have to get [two or three] buses and then it's quite a long way to walk there as well, it's not easy. (P20_female_Pakistani Muslim_58 years)

#### 3.3.3 Economic exclusion

In addition to experiences of financial precarity presented earlier, the loss of occupation or ability to engage in the labour market amongst working age participants was a further cause and consequence of exclusion from society, as well as being a compounding factor due to the associated mental health problems:

The loss of that job was terrible, more than the loss of income, it was the psychological impact of that … Social isolation is brought about on a day‐to‐day basis because of the lack of income, because it's very costly going out and about, being involved in things… (P16_male_White British_57 years)

There were also instances where some participants, for different reasons, found that they had to self‐exclude themselves fully or partly from the workplace:

I'm obese and … then I was bullied in work and I stuck it out to six months and the pressure just I think got too much for me and I became really unwell … And then in the end … I was diagnosed with deep depression… (P05_male_South Asian_37 years)

Being unable to engage in paid employment was viewed as a negative consequence of ill health. Some participants perceived themselves to be trapped in a vicious circle, struggling with the balance that had to be struck between effective self‐management and having the capacity to work, as explained below:

…there's this weird setup, especially with the benefits system where, if you're doing something to help yourself, they see that as evidence that you can do stuff, you know, you can go out and work. But if you go out and work then you don't have the energy to do the self‐management, and if you don't do the self‐management, you don't have the capacity to go out and work. (P12_male_white British_52 years)

#### 3.3.4 Health‐related stigma

Some participants spoke about trying to cover up or hide their health conditions and these narratives were found largely amongst minority ethnic participants. The reasons provided varied but for most participants, these were due to feelings of being judged or stigmatised and could negatively impact upon self‐management practices:

…when I first had epilepsy I would actually hide. People's attitudes have changed a little bit but not much. I was very self‐conscious about having seizures and I used to go and actually sit in a public … bathroom on the toilet… (P07_female_Indian_49 years)

Another participant spoke about continuing to hide her diagnosis from her employer which originally stemmed from historical perspectives about mental ill‐health:

…I have never told my job [about being bi‐polar] I kept that from them … I've been there for nearly 20 years. And at that time it was different … back then it was like ‘Oh you've got this, oh, you can't do that then’. And I've shown that myself that I can do it. Just because you've got this, doesn't mean that you're, you know, a freak… (P14_female_British Asian_57 years)

A further participant explained that due to his parents being of a different generation of South Asians, they failed to view his depression as a serious illness which impacted on his self‐esteem:

But that community, that generation of people, it was completely different like the way they were brought up, integration within Britain, so I think it's been a challenge no doubt. Like even to explain to them, like what health conditions are, what depression is. They've said depression isn't a serious illness. (P04_male_British South Asian_26 years)

### 3.4 Adapting self‐management strategies

#### 3.4.1 Cost‐free and low‐cost activities

Like others who manage MLTCs, people experiencing socioeconomic deprivation also engaged in physical activity, tried to improve their diet, and sought out complementary/alternative treatments. Some participants pursued activities through community‐based organisations or charities:

…they have a local [diabetes] group in the local Church and I go there and … we're quite lucky because actually they do kind of give you more of a type of food that you can eat whilst you're there. So sometimes I can manage to have something to eat… (P05_male_South Asian_37 years)

Resource constraints could however impact on self‐management opportunities. Some participants were unable to try alternative treatments and despite believing things like acupuncture or massage would be beneficial; these were not an affordable option:

…things like acupuncture people say sometimes helps them but I just can't afford them. There's so many things I would love to try but I just can't—they're just outside of my financial ability to pay for because I have no saving… (P22_male_White British_35 years)

Swimming was a popular form of physical activity liked by participants, describing the water as giving a feeling of weightlessness which could help alleviate chronic pain. Some participants would use their benefit payments to access sporting activities:

…the swimming is just like really healing … I bought a [swimming] season ticket with my ESA money, my PIP money … When I don't swim, I can feel [the pain] even more. It's my only … where I can move my body and not feel pressure… (P15_female_European‐North African_50 years)

Some participants engaged in free or low‐cost self‐management practices at times, others described the financial outlay of facilitating self‐management leading to making sacrifices elsewhere:

…if you take the gym for example, and they've suddenly increased the price, and I'm like, ‘Oh, shall I cancel it?’ But I thought, no. I mean, I really need to cancel it, but I thought I'll rather sacrifice other things [like supplements and alternative treatments] and keep it going… (P09_female_Black African_56 years)

Due to financial constraints, some participants incorporated physical activities into daily routines which was cost‐free:

So housework is great, dusting, you're reaching … you're stretching. The washing up … I sit down a lot because of the oxygen in the legs business, but it's a physio exercise without it being a physio exercise … [I] switch off when a physiotherapist says here's a list of exercises, and I'll look at them and think, how can I incorporate that into something that I do in my daily activities? … when I'm waiting for a bus, I'll start doing these stretching exercises… (P16_male_White British_57 years)

#### 3.4.2 Cultural and lifestyle appropriate strategies

Accessing culturally appropriate facilities and food was an issue for several minority ethnic participants. Challenges surrounding affordability of provision, acted as a further barrier to self‐management:

…to keep healthy, I wanted to … start swimming, but where our area is, it's terrible, right. Number one they don't have no facilities, right, for moral Asian women to swim. Because I don't like to go in mixed pools… (P20_female_Pakistani Muslim_58 years)

Some participants cooked fresh food to maintain a healthy diet and support self‐management, a choice that could be connected to cultural background. Some guidelines, such as those related to diabetes, did not fit into people's way of life, referring to their food choices as their lifestyle:

The other thing though is, with the diabetes, yeah, the thing is you want to eat sweets, you want to eat cake, you want to eat this, you want to eat a bit more, you know, it's a lifestyle, you want your bit of fried food. So it's difficult to maintain. It's difficult to get the right support. (P14_female_British Asian_57 years)

Some participants reported seeking alternatives to traditional medication or to use these alongside them. In some cases, minority ethnic participants made a connection between the use of alternative medicines and their cultural upbringing/background:

I think because of the kind of, the ethnic background that I've got, we already have our own type of herbal type cures and things that we can use. So I always feel as though I've got a little bit more of a toolkit in comparison to I think people who don't have a background with a different form of medicine. (P05_male_South Asian_37 years)

## 4 DISCUSSION

The findings suggest a myriad of intersecting factors relating to MLTCs and socioeconomic disadvantage, demonstrating the compounding effect these have on quality of life and participation in society. Self‐management makes people increasingly accountable for their own health, placing demands on the individual. While the Burden of Treatment Theory shines a spotlight on a shift towards self‐management, for example, through exploring the relationship between a person's ill‐health, social capital, and healthcare services,^8^ self‐management of MLTCs is not the sole responsibility of the individual. Rather, ‘it requires a collaborative approach in which the health‐care system delivers on‐going support’ for people managing their own conditions.^21^ Current challenges in general practice are, however, said to be dominated by social rather than medical needs, reflecting the need for an intersectional approach to healthcare.^26^

Health literacy refers to a person's ability to access, understand and use information to make decisions about their health.^27^ Low health literacy results in challenges around making informed decisions about health and has the potential to reinforce existing health inequalities.^6^ Low health literacy was evident amongst participants, alongside low financial literacy; the latter being a life skill highly related to the well‐being of individuals and crucial to financial inclusion.^8^, ^28^ Health‐related financial assistance is designed to ease the financial strain of everyday life, yet the structures in place can exacerbate symptoms of mental health, leading to the worsening of physical health in some cases.^29^ The challenges experienced by participants' trying to access health‐related state benefits, contributes to our understanding of how the structures shaping the healthcare and state welfare systems intersect. As such, if self‐management is to reduce engagement with formal health services, as it is intended, investment is needed to support individuals to acquire the skills necessary to navigate systems at a societal level and use the individual resources available to them.^8^

Correspondingly, the findings highlight challenges amongst participants around asserting candidacy, which is how people define their eligibility to access a particular service.^30^ Candidacy can dictate help‐seeking behaviours,^31^ as seen through the communication challenges that some minority ethnic participants faced, as well as more general health service challenges around time‐limited communications and lack of consistency. Addressing inequalities in access to healthcare requires recognition that the distribution of staff in practices, especially those in deprived areas, is equitable rather than equal and reflects population need.^26^ However, while previous research suggests that HCPs are integral to assisting with socioeconomic issues that impact upon health, strategies of support are based more on individual decisions by practices rather than through systems‐level and structural changes.^26^

Obtaining health‐related information relies on adequate health literacy but as the development of digital health services grows, the skillset required for accessing such services becomes more complex,^32^ creating an even greater need for effective strategies of support. The ability to process information and engage in digital services as well as having access to technology are some of the key domains necessary to use digital health resources; a deficit in any area can cause health inequalities.^33^ Low digital literacy amongst participants revealed challenges around self‐management due to limited digital skills/education and limited access to digital resources because of financial restrictions. Difficulties (including financial restrictions) in accessing digital/electronic health information intersect with experiences of low health and financial literacy, but also with ethnicity since participants from minority ethnic backgrounds were evidenced as less technologically able to self‐educate or access health apps. While electronic health (eHealth) has the potential to improve health outcomes, there are concerns that the eHealth field is not equitable and more must be done to match the needs of its users when developing interventions for socioeconomically disadvantaged groups.^34^ Policy approaches to the development of digital interventions have been criticised for ignoring the complexity and intersectionality of digital inequalities. Our findings support the view that greater emphasis is needed on addressing the deep‐seated issues of structural inequalities in society, especially if digital technology in healthcare is to continue to grow.^35^

Access to social capital is also of great importance for utilising healthcare services and supports individuals' capacity to manage their conditions. While access to socioeconomic resources can limit health options, a lack of social capital affects the extent to which individuals and their networks can capture, possess and mobilise social resources while also trying to navigate the structures around them.^8^ Many participants reported low social capital which resulted in experiences of isolation and/or loneliness which worsened mental health. Individuals who are socially excluded are found to lack important connections to family, friends and neighbours and typically have weaker social ties and lower social capital.^36^, ^37^ Participants with access to informal support networks, were, however, able to self‐manage better. A nuanced relationship exists between social capital and health and may vary according to certain physical health conditions.^38^ Feeling lonely has been linked to early mortality^39^ and existing interventions to address social isolation are typically aimed at supporting older people due to their increased risk of loneliness/isolation.^40^ Most participants were of working age but could not engage in the labour market, leading to both economic and social exclusion. The nature and causes of feelings of social isolation and/or loneliness amongst younger adults can be distinct from those experienced by older adults. To address low social capital amongst younger adults with MLTC who experience socioeconomic disadvantage, there is a need for interventions aimed at reducing loneliness to not only increase social contact but also consider other social determinants of health, such as access to adequate housing.^41^

Finally, participants from minority ethnic backgrounds discussed stigma relating to their health conditions which impacted self‐management. The ‘socio‐historical’ context associated with different ethnic backgrounds impacts on individual experiences as well as their social positioning.^42^ The process of supressing or hiding symptoms of health in this context has been termed ‘silent suffering’ due social unacceptability of being unwell^30^ and can be a barrier to help‐seeking behaviours. Stigma and inequality are interrelated concepts that are strongly linked to how people respond to health threats, and hiding symptoms can become a coping mechanism to manage such threats.^43^ The findings suggest that people with MLTCs who experience additional inequities due to socioeconomic deprivation and ethnicity may be more likely to experience compounding stigma. Being aware of this phenomenon, and how it can become ingrained in people who then internalise how society devalues them, is important for addressing health inequalities and developing tailored interventions.^44^ Antistigma campaigns/strategies tailored to specific ethnic backgrounds and conditions are therefore considered to be one beneficial way of improving health outreach.^45^

## 5 IMPLICATIONS FOR POLICY AND PRACTICE

The additional inequalities associated with MLTCs compared to single LTCs call for a response from within the health system to both strengthen generalist skills alongside specialist skills and consider the intersectionality of key socioeconomic factors.

As such, there is a need for policymakers to invest in supporting socioeconomically disadvantaged populations to access digital health resources and health‐related financial assistance, which should include support at the societal level to navigate complex systems. Policy and practice also need to address health‐related stigma, particularly for mental illness amongst minority ethnic populations which can be worse for those with MLTCs. Antistigma strategies are needed at the community level to promote culturally sensitive communications about health concerns. Antistigma strategies and education are also needed to increase cultural awareness across primary care.

To address these structural inequalities in health and society, policy makers and practitioners must take a holistic approach to the issues of health literacy, digital exclusion and health‐related stigma. Doing so will help enable tailored interventions to acknowledge the intersecting dimensions surrounding health inequalities. More research on how these changes may be achieved at both a micro and macro level is needed, especially in relation to the roles of extended primary care teams and social prescribers.

## 6 STRENGTHS AND LIMITATIONS

The main strength of the study is the ethnic and age diversity of the sample, which has enabled the presentation of data from people who are typically underrepresented in research on MLTCs. These are important findings since most previous studies focusing on MLTCs are with older, White British people. The integration of an intersectional approach to data analysis has provided a holistic understanding of the challenges faced by individuals with MLTCs. The timely nature of the study provides in‐depth insights into the early health implications of the cost‐of‐living crisis. A potential limitation of the study is that the findings are of direct relevance within a UK context and may not necessarily be transferable to other geographic settings. The study also provides insights into participants' experiences during a particular point in time and as such, it is not possible to say how these experiences will evolve over time.

## 7 CONCLUSION

This paper explored individual experiences of socioeconomic deprivation and their impact on self‐management of MLTCs and reveals complexities surrounding access to healthcare services and financial assistance, and how digital health has potential to improve effective self‐management of MLTCs. Access to technology and ways to enhance digital skills need addressing to reduce exclusion to self‐management. The stigma associated with health conditions amongst minority ethnic groups indicates the compounding effects of multiple inequalities. Ultimately, this study has emphasised the importance of raising awareness in practitioners and commissioners of the complexities surrounding the lives of people experiencing socioeconomic deprivation, and the need for targeted strategies to promote self‐management of MLTCs.

## AUTHOR CONTRIBUTIONS

**Abi Woodward**: Writing—original draft; writing—review and editing; methodology; formal analysis; conceptualisation. **Danielle Nimmons**: Writing—review and editing. **Nathan Davies**: Writing—review and editing. **Kate Walters**: Writing—review and editing. **Fiona Stevenson**: Writing—review and editing. **Joanne Protheroe**: Writing—review and editing. **Carolyn A. Chew‐Graham:** Writing—review and editing. **Megan Armstrong**: Writing—review and editing; supervision; funding acquisition; conceptualisation.

## CONFLICT OF INTEREST STATEMENT

Professor Chew‐Graham is Editor in Chief of Health Expectations. The authors declare no conflicts of interest.

## ETHICS STATEMENT

Ethical approval was granted by the Health Research Authority (HRA) (22/LO/0227) and the University College London (UCL) Research Ethics Committee (22357/001).

## Supporting information

Supporting information.

## Data Availability

Research data are not shared.
