# Supplementary material for: A qualitative exploration of the barriers and facilitators to self‐managing multiple long‐term conditions amongst people experiencing socioeconomic deprivation
Source: Health Expect. 2024 Apr 16;27(2):e14046. doi: 10.1111/hex.14046 (PMC11019445; doi:10.1111/hex.14046)
Supplement: Supplementary file 1 — Supporting information. [file HEX-27-e14046-s001.docx]

**Appendix 1: Topic Guide**

**Background**

1. ***Ice breaker* - Can you begin by telling me more about your health conditions?**

- Recap/clarify type of conditions (if unclear), how long ago were you diagnosed?

1. **Thinking about how you manage your health conditions day-to-day, what does a typical week look like for you?**

- Do you have a routine? (visits from carer/family, housework, childcare, going to work, exercise, routine healthcare/physio appointments, social groups…)
- Unless living alone, ask about role of other household members (care givers?)

1. **Can you tell me about anything significant that you struggle with because of your health conditions?**

- Any specific challenges related to having more than one health condition?
- Mental health (anxiety, depression…), lifestyle/quality of life, poor sleeping.
- Social restrictions/interactions, impact on quality of relationships with others
- Practical things inside and outside the home, physical activity
- Nutrition, eating healthy diet, affordability of food
- Unable to work, financial restrictions…
- Work-life balance i.e. struggle to attend appointments, find time to cook/eat healthily

1. **In what ways does your financial situation, if at all, impact your ability to manage your health conditions? *(Draw upon previous responses if needed)***

- Benefits e.g. struggle with renewals and if so, does anyone help with this? OR if they aren’t claiming benefits – why? (e.g. doesn’t know how to or what entitled to + other reasons?)
- Heating the home/eating healthily/anxiety over money and paying bills /digital access…
- If working – self-isolating during pandemic (loss of earnings)

**Health literacy**

1. **Do you feel you have been told enough about your health conditions?**

- Good understanding of conditions to make decisions about health? (same for both/all conditions?)
- Knowing where/how to get info if needed/had a concern (how is info accessed and is it the same for both/all conditions?) - Who provides you with info – HCP, family, friends, internet?
- Opportunity to speak to a health care professional if you have any questions/queries?
- Barriers - Difficulties understanding health information
- Barriers - Don’t know who to talk to or can’t access anyone / language/cultural / online vs. paper / No internet / Don’t know where to look / Can’t navigate the system / difficulties filling out forms…
- How might your access to info/resources be improved?

1. **Can you tell me about a specific time when you had a particularly good or not so good experience during an appointment with your GP or another health care professional?**

- Feel you are being listened to or not? (paying attention to you…)
- Feeling of trust?
- Easy to follow advice (if so, how often do you follow their advice?)
- Being told about different health services available and when to use them?
- Lacking control over own life?
- Telephone consultations vs. face-to-face (pre/post-covid)
- Is there anything that would improve your experience? e.g. more continuity/coordination between GPs/health services? Easier access to info/easier to understand info?

**Support and self-management**

1. **What sorts of things do you do yourself which help you manage your health conditions?**

- Monitoring – recognising if things are getting better or worse (diary, apps…)
- Asking for help/seeking out information (HCP, friends, family?)
- Use of technology/online services e.g. to monitor pain management, anxiety (apps, ‘wearables’),
- Changes made that have a positive impact on overall health/mental health e.g. trying to eat healthily? Cutting down on things that worsen health condition/s? Doing exercises/physio/ walking, gardening…
- If made changes, what prompted this e.g. advice from friend/professional/internet?
- Anything that makes monitoring/managing health difficult e.g. housing conditions (damp, cold, disrepair)?
- Anything you feel you would like to do or ought to be doing? (Probe about any additional lifestyle changes if not already covered).

1. **What support are you receiving to help you manage your health conditions?**

- Help with what specifically (medical and non-medical)
- Support from who? e.g. HCP, GP practice/LA, housing Assoc, benefits, family, friend, neighbour…
- Adequate for both/all health conditions?
- **Current and past support** - any changes with the type/level of support received?
- Use of online services/resources? (Examples)
- Housing issues? i.e. addressing damp/ disrepair/ accessibility (LA, housing assoc., private landlord…)
- **If none...** why not/ barriers (language, cultural, access difficulties/visual impairment, internet…)
- **Improvements** needed? e.g. any specific support/element of support that you have tried to access but struggled with? Issues accessing technology/online services?)

**Accessing support with the locality and wider area **(Refer to responses from Q8 if needed)****

1. **In terms of where you live (local area), can you tell me about any difficulties you have had, or continue to have, with accessing the support or health services you need to manage your conditions?** Good/poor local access to health services e.g. GP surgery, pharmacy, hospital?

- Telephone vs. face-to-face appointments (or lack of appointments)
- Digital/technological barriers e.g. booking online/through an app
- Affordable transport links…
- Cultural/language barriers?
- Lack of local knowledge – new to the area/not sure where to look…
- What **improvement** could be made to help you?

1. **Can you tell me about anything you have been referred to or found yourself to help you manage your conditions (or that help in general with your health and wellbeing)?**

- **What?** e.g. community groups, pain support groups/therapy/massage/meditation/social activities (social café, dementia café, lunch clubs, singing, walking groups, social prescribing services…)
- **Where?** Local area or had to travel further afield?
- **How?** e.g. self-referral or by HCP? Difficulties with referral process?
- **If none** – have you accessed anything in the past and if not, why not? (nothing appeals/local services have been cut/lack of investment in area / Language/cultural barriers?)
- What **improvements** could be made to help you?

**Covid-19 - Impact on long-term conditions**

1. **How has the Covid-19 pandemic impacted on your ability to manage your health conditions?**

- Access to support services (restrictions/lockdown/closure of buildings…)
- Social isolation/loss of social interaction (needing to self-isolate?)
- Reduced physical activity
- Loss of support from carer/other people
- Loss of patient transport
- Interruptions to health care e.g. cancelled appointments, unable to see GP…
- Lasting effects of pandemic - overall deterioration of mental health/health and wellbeing/accelerated existing problems…‘Long Covid’?
- Positive? More regular contact over the phone, people coming to you e.g. doorstep visits, phone calls, remote social activities, packs posted out i.e. arts and craft activities…)
- If working – self-isolating and loss of earnings

**Closing question**

**12. In an ideal world, what three things do you think would enable you to manage your health conditions better?**
